# 272. Do Empiric Antibiotics Improve Outcomes in Clinically Stable Patients Admitted with COVID-19 Pneumonia? Retrospective Cohort Study of 221 U.S. Hospitals, March 1st, 2020-December 31st, 2020

**DOI:** 10.1093/ofid/ofac492.350

**Published:** 2022-12-15

**Authors:** Alexander Lawandi, Jeffrey R Strich, Xioabai Li, Christina Yek, Sarah Warner, Sameer S Kadri

**Affiliations:** McGill University Health Centre, Mont Royal, Quebec, Canada; Critical Care Medicine, National Institutes of Health Clinical Center, Bethesda, Maryland; National Institutes of Health, Bethesda, MD; National Institute of Allergy and Infectious Diseases, Bethesda, Maryland; Critical Care Medicine, National Institutes of Health Clinical Center, Bethesda, Maryland; National Institutes of Health Clinical Center, Bethesda, Maryland

## Abstract

**Background:**

Patients admitted with COVID19 pneumonia often receive initial empiric antibacterial therapy (IEAT) despite a known low probability of bacterial co-infection. However, evidence supporting this practice is lacking. We studied the impact of IEAT on the risk of in-hospital mortality, clinical deterioration and antibiotic-associated risks in stable inpatients with COVID-19.

**Methods:**

Adult inpatients coded for COVID-19 pneumonia stable (no mechanical ventilation or vasopressors) on admission (+1 day) without a clear indication for antibiotics, were identified at hospitals in the Premier Healthcare Database. Patients who received IEAT, defined as the receipt of ≥ 1 antibacterial agent on admission (+1 day), were compared to a control group, using binomial regression with overlap weight matching and downstream adjustment for baseline characteristics (age, gender, race, admission month, surge index, Elixhauser score, any AOFS organ failure POA, ICU admission on day 0 to +2, receipt of remdesivir, corticosteroids, and tocilizumab). The primary outcome was in-hospital mortality or discharge to hospice; secondary outcomes included need for mechanical ventilation on day2+, and rates of non-POA-acute kidney injury (AKI).

**Results:**

At 221 hospitals between March–December 2020, 39,517 (74%) of 53,431 stable COVID-19 admits received IEAT. Patient and encounter characteristics are shown in Table 1. The crude mortality rates were 12.2% in IEAT recipients and 10.9% in controls. In adjusted analysis of patients who survived beyond admission day, mortality was 11.57% (95% CI 11.24-11.90%) in IEAT recipients and 11.23% (95% CI 10.72-11.74) in controls, for a difference of 0.34% (95% CI -0.23-0.91%, p = 0.24). Subsequent mechanical ventilation occurred similarly between groups (5.72% vs. 5.77%, p=0.83). The adjusted rate of AKI was 2.47% (95% CI 2.31-2.64%) in IEAT recipients, and 3.04% (95% CI 2.74-3.35%) in controls, for a difference of -0.57% (95% CI -0.92-0.22%, p = 0.0014).

**Table 1.**
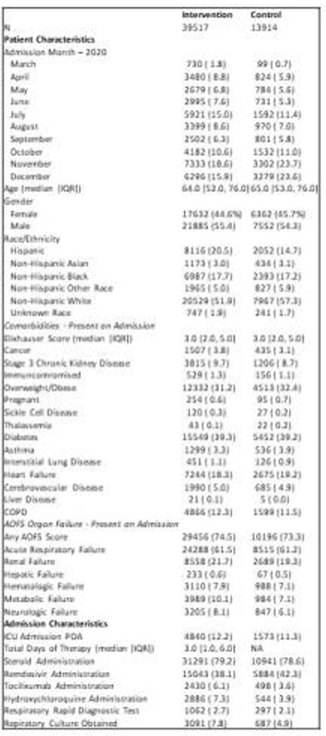
Demographics, clinical and hospital characteristics for patients treated with initial empiric antibiotic therapy (intervention) versus those not treated (control).

**Figure 1.**
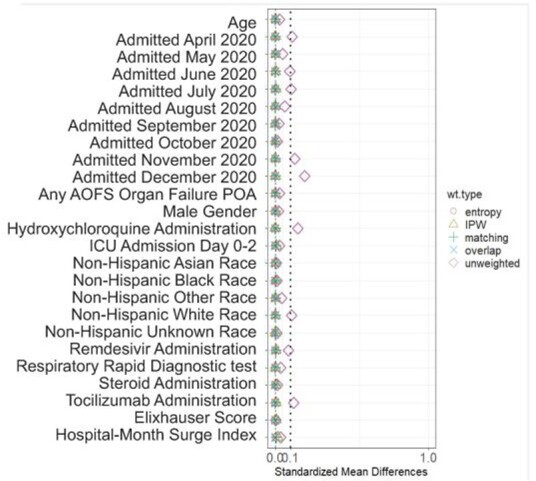
Standardized mean differences in included covariates before and after several matching strategies comparing covariate values for patients treated and not treated empirically with antibiotics

**Conclusion:**

In patients with COVID19 initially admitted to the ward, IEAT was not associated with a reduction in mortality or deterioration requiring mechanical ventilation, but with a clinically insignificant reduction in AKI. Empiric antibiotics can likely be safely withheld in this population.

**Disclosures:**

**All Authors**: No reported disclosures.

